# Complete Genome Sequences of Three Phocaeicola vulgatus Strains Isolated from a Healthy Japanese Individual

**DOI:** 10.1128/mra.01124-21

**Published:** 2022-02-03

**Authors:** Hanh Vu, Yoshinori Muto, Masahiro Hayashi, Hideki Noguchi, Kaori Tanaka, Yoshimasa Yamamoto

**Affiliations:** a United Graduate School of Drug Discovery and Medical Information Sciences, Gifu University, Gifu, Japan; b Institute for Glyco-core Research (iGCORE), Gifu University, Gifu, Japan; c Life Science Research Center, Gifu University, Gifu, Japan; d Joint Support-Center for Data Science Research, Research Organization of Information and Systems, Mishima, Japan; University of Arizona

## Abstract

Phocaeicola vulgatus (formerly Bacteroides vulgatus) is a pathogenic anaerobic bacterium frequently involved in human infections. We present the complete genome sequences of three *Phocaeicola vulgatus* strains isolated from the same healthy person, determined by hybrid assembly using Nanopore long-read sequencing and DNBseq short-read sequencing.

## ANNOUNCEMENT

Phocaeicola vulgatus, one of the most numerically predominant *Bacteroides* species, has been extensively studied for its critical roles in infectious diseases as well as in other aspects of human health (1–4). Despite their clinical importance, *Bacteroides* species are most likely underrepresented in public genome databases, especially in terms of complete genomes of strains isolated from humans. Thus, there exists an urgent need for high-quality, well-established genome data to support studies on microbiome diversity and function. Hence, we sequenced and assembled the complete genomes of three *P. vulgatus* strains isolated from a healthy Japanese volunteer.

The fecal sample was collected from a volunteer who had normal bowel activity and no history of antibiotic use during the 3 months prior to the study. Samples were collected with Puritan fecal Opti-Swab CB-206 and cultured in an anaerobic atmosphere on *Bacteroides* bile esculin (BBE) agar (Kyokuto) and BBE with ceftazidime (30 mg/L) for 48 h at 37°C. Colonies of more than 1-mm diameter were subcultured on Gifu anaerobic medium agar (Nissui) under the same conditions. The isolates were identified using matrix-assisted laser desorption ionization–time of flight mass spectrometry (MALDI-TOF MS) (MALDI Biotyper [MBT]). *P. vulgatus* strains were incubated in ABCM broth (Eiken Chemical) in an anaerobic chamber (Hirasawa) at 35°C for 12 h. Bacterial pellets were obtained by centrifugation. Total DNA was extracted using a NucleoBond high-molecular-weight (HMW) DNA kit (Macherey-Nagel). The DNA was quantified using Qubit double-stranded DNA (dsDNA) high-sensitivity (HS) assay kits (Thermo Fisher) and qualified with a NanoDrop instrument (Thermo Fisher) at an optical density of 260/280 nm (OD_260/280_), and fragments were checked by electrophoresis. The same qualified DNA templates were used for both long-read and short-read sequencing.

Genome assembly was conducted using a hybrid approach combining Nanopore long-read sequencing and DNBseq short-read sequencing, similar to a previously described method (5). For long-read sequencing, a library was constructed using a ligation sequencing kit (SQK-LSK-109; Oxford Nanopore Technologies [ONT]), and sequencing was performed using a GridION X5 system (ONT) on a FLO-MIN106 flow cell. Long-read sequence data were base called using Guppy v.4.2.3. The raw reads were subjected to trimming and quality filtering using Porechop v.0.2.4 (https://github.com/rrwick/Porechop) and Filtlong v.0.2.0 (minimum length 100 bp) (https://github.com/rrwick/Filtlong). For short-read sequencing, the MGIEasy FS DNA library prep set (MGI Tech) was used for library construction. Subsequently, 2 × 150-bp paired-end sequencing was performed using the DNBSEQ-G400 platform (MGI Tech). The raw sequencing reads were processed using fastp v.0.20.1 (6) for trimming adapters and low-quality data, and approximately 3.5 million read pairs (1.0 Gbp) were sampled using SeqKit v.0.16.1 (7). High-quality short-read and long-read sequences were assembled using Unicycler v.0.4.8 (8) with default settings. The assembled contig graph was confirmed using Bandage v.0.8.1 (9), and the integrity of the assembled genomic data was confirmed using CheckM v.1.1.3 (10). Annotation of the assembled genomes was performed using the DDBJ Fast Annotation and Submission Tool (DFAST) (https://dfast.nig.ac.jp/). Default parameters were used for all software unless otherwise specified.

The genome information is summarized in Table 1. According to CheckM, all three of the obtained genomes were 99.25% complete with no contamination.

**TABLE 1:** Information of the complete genome sequences of three *P. vulgatus* strains isolated from a healthy Japanese individual

Parameter^*a*^	Data for strain:		
	MG01-03	MG01-07	MG01-10
DNBseq sequencing			
No. of reads	32,198,982	17,338,628	28,459,232
Size (kb)	4,829,847	2,600,794	4,268,884
Avg coverage (×)	911	490	805
DRA accession no.	DRR328570	DRR328571	DRR328572
ONT sequencing			
No. of reads	133,757	175,618	123,967
Size (kb)	1,129,693	1,170,228	1,148,433
Avg read length (bp)	8,445	6,663	9,264
Avg coverage (×)	213	221	217
DRA accession no.	DRR328574	DRR328575	DRR328573
Assembly			
Genome structure	1 chromosome and 2 plasmids	1 chromosome and 4 plasmids	1 chromosome and 4 plasmids
DDBJ/GenBank accession no. (chromosome/plasmid name)	AP025232 (GAIMETA0103)	AP025235 (GAIMETA0107)	AP025240 (GAIMETA0110)
	AP025233 (pMG01-03_1)	AP025236 (pMG01-07_1)	AP025241 (pMG01-10_1)
	AP025234 (pMG01-03_2)	AP025237 (pMG01-07_2)	AP025242 (pMG01-07_2)
		AP025238 (pMG01-07_3)	AP025243 (pMG01-10_3)
		AP025239 (pMG01-07_4)	AP025244 (pMG01-10_4)
Genome size (bp) (chromosome/plasmid name)	5,073,274 (GAIMETA0103)	4,985,319 (GAIMETA0107)	4,957,169 (GAIMETA0110)
	5,594 (pMG01-03_1)	7,659 (pMG01-07_1)	7,659 (pMG01-10_1)
	4,306 (pMG01-03_2)	5,594 (pMG01-07_2)	5,594 (pMG01-10_2)
		4,306 (pMG01-07_3)	4,306 (pMG01-10_3)
		2,784 (pMG01-07_4)	2,784 (pMG01-10_4)
G+C content (%) (chromosome/plasmid name)	42.4 (GAIMETA0103)	42.3 (GAIMETA0107)	42.2 (GAIMETA0110)
	39.6 (pMG01-03_1)	37.8 (pMG01-07_1)	37.8 (pMG01-10_1)
	42.9 (pMG01-03_2)	39.6 (pMG01-07_2)	39.6 (pMG01-10_2)
		42.9 (pMG01-07_3)	42.9 (pMG01-10_3)
		41.5 (pMG01-07_4)	41.5 (pMG01-10_4)
No. of coding sequences^*b*^	4,552	4,484	4,454
No. of RNAs^*b*^	102	108	108

a DRA, DDBJ Sequence Read Archive.

b DFAST, DDBJ Fast Annotation and Submission Tool.

### Data availability.

The complete genome sequences of the three *P. vulgatus* strains are available in DDBJ/EMBL/GenBank under the accession numbers listed in Table 1.
